# Diversity and drivers of arbuscular mycorrhizal fungi in the rhizosphere soil of wine grape in the eastern foot of Helan Mountain in Ningxia of China

**DOI:** 10.3389/fmicb.2025.1700411

**Published:** 2025-10-29

**Authors:** Qiangqiang Zhang, Siyuan Ma, Ruotong Wang, Ling Li, Qingchen Zhang, Mingxiu Ju, Peiwen Gu

**Affiliations:** ^1^School of Forestry and Protoculture, Ningxia University, Yinchuan, China; ^2^School of Agriculture, Ningxia University, Yinchuan, China; ^3^Department of Pharmacotherapy and Translational Research, University of Florida, Gainesville, FL, United States

**Keywords:** arbuscular mycorrhizal fungi (AMF), wine grape, colonization status, diversity, soil factors

## Abstract

**Introduction:**

Arbuscular mycorrhizal fungi (AMF) are symbiotic microorganisms that exert positive effects on their host plants. However, their colonization and community diversity in wine grapes remain unclear.

**Methods:**

This study investigated roots and rhizosphere soils from Cabernet Sauvignon grapevines in vineyards in seven ecological regions at the eastern foot of the Helan Mountains in Ningxia, China. We employed Illumina MiSeq high-throughput sequencing to analyze AMF community composition and diversity in the rhizosphere soil, and examined the effects of soil factors on AMF communities.

**Results:**

The results showed that the grapevine root system was colonized by AMF, with significant spatial heterogeneity in colonization rates and spore densities across the sample plots. Differences in the diversity of the AMF communities in the rhizosphere soil of wine grapes in the different sample plots were observed, and these AMF communities were further divided into three groups. In total, 168 operational taxonomic units were detected in the rhizosphere soil, corresponding to 40 AMF species from five orders, seven families, and seven genera. The *Glomus* and *Glomus melanosporum* were the dominant genus and species, respectively. *Claroideoglomus* and *Glomus* were identified as biomarkers. Soil pH and organic matter were key factors influencing AMF colonization, abundance, diversity, and community composition.

**Discussion:**

The grape rhizosphere in this region hosts a rich diversity of AMF. This finding provides a reference for the protection and commercial cultivation of AMF in wine grape rhizospheres.

## 1 Introduction

Arbuscular mycorrhizal fungi (AMF), obligate symbiotic fungi belonging to the phylum Glomeromycota and Mucoromycota (Lutz et al., 2025), are a crucial group of soil microorganisms that are widespread in natural ecosystems (Liang et al., 2022; Xu et al., 2022). AMF make symbiotic associations with more than 80% of terrestrial plant roots (Luginbuehl et al., 2017; Liao et al., 2018; Ma et al., 2022). In this relationship, AMF obtain photosynthetic metabolites from plants and improve water and mineral nutrient uptake (Mickan et al., 2016; Xu et al., 2022) enhancing plant adaptability (Shaul-Keinan et al., 2002). Considering their ecological significance and the pervasive occurrence of their symbiotic associations, elucidating the factors that modulate AMF community composition is pivotal to understanding their distribution patterns and optimizing their ecological functions.

AMF community composition is affected by geographical area (Zhang et al., 2021), environmental conditions (Jansa et al., 2014; Yang et al., 2016), management measures (Geel et al., 2015), host plants (Vályi et al., 2014) and other factors. At local scales, host plants and environmental factors are the primary drivers of AMF community formation. While differences in host plants at the species or genotypes influence the structure of AMF communities (Zeng et al., 2023), studies have found that environmental factors have a greater impact on root AMF community structure. Among these, soil pH is considered one of the factors most closely related to the root AMF community (Han et al., 2023; Xu et al., 2023). In addition, soil chemical factors such as total nitrogen (Han et al., 2020; Lu et al., 2022), organic carbon (Garo et al., 2022), total phosphorus (Zhang M. et al., 2022), available potassium (Wang et al., 2024), available phosphorus (Garo et al., 2022; Xu et al., 2023); climatic characteristic factors such as precipitation (Li et al., 2021); and geographical factors, such as altitude (Rasmussen et al., 2022) and slope direction (Wang et al., 2022) have been reported to affect the AMF community structure of roots. These studies underscore the complexity of the influence of soil factors on the formation of rooted AMF communities, highlighting the need to distinguish between ecosystems for in-depth analysis.

Grapes (*Vitis vinifera* L.) are a globally cultivated perennial fruit crop prized for their high nutritional value and extensive history of cultivation (Massa et al., 2020). Investigations on grapes are primarily concentrated on postharvest technology (Mencarelli and Bellincontro, 2020), disease and pest management (Girardello et al., 2019), terpenoids (Mele et al., 2021), variety breeding (Fernández-López et al., 2022) and wine aroma (Liu et al., 2017). However, the exploration of beneficial microbial resources, particularly the diversity of symbiotic AMF, remains limited. Schreiner and Mihara (2009) demonstrated the stability of *V. vinifera* root AMF diversity in Oregon vineyards across seasons, with observed variations based on soil type and vine age. Subsequent research further elucidated the influence of soil depth, management zones, and timing of the growing season on the grape root AMF community (Schreiner, 2020). However, studies specifically targeting AMF community diversity associated with Chinese wine grapes remain notably scarce.

The eastern foot of the Helan Mountains (EFHM) in Ningxia is a prominent geographical indication product protection area for wine in China (Zhang Z. et al., 2022). Located in the global “golden zone” for grape cultivation, this region boasts significant international acclaim (Hu et al., 2021). Fang et al. (2011) investigated the spatial distribution of root AMF in *V. vinifera* cv. Cabernet Sauvignon across five distinct vineyards in northwest China. Their findings confirmed the beneficial symbiotic relationship between grapes and AMF, with *Glomus* species being dominant. Common species such as *G. constictum*, *G. intraradices*, *G. mosseae*, and *G. versiforme* and rare species such as *G. claroideum*, *G. coronatum*, *G. etunicatum*, *G. luteum*, *A. rehmii*, and *A. rugosa* haved been identified in the rhizosphere of grapes in the EFHM. Zhang et al. (2025) observed differences in the diversity and composition of rhizosphere soil AMF communities among different wine grape varieties in the EFHM. Specifically, they observed the richest AMF community composition for Cabernet Sauvignon. Nevertheless, comprehensive assessments of AMF colonization and community composition in the root system and rhizosphere soil across different wine grape ecotopes in this region remain unexplored. Our study applied high-throughput sequencing to investigate the rhizosphere soils of *V. vinifera* cv. Cabernet Sauvignon grapes in various ecological regions within the EFHM. We classified and identified the isolated AMF spores based on their morphological characteristics. The acetic acid-ink staining method was used to observe the AMF infection status in grapevine roots. Specifically, the objectives were to explore the colonization patterns of AMF in wine grape roots, analyze AMF diversity in wine grape rhizosphere soil, elucidate the community composition and distribution of AMF in wine grape rhizosphere soil, and examine the relationships between AMF community diversity and soil factors. This study lays the groundwork for maximizing the utilization of AMF resources and the development of specialized microbial fertilizers tailored to wine grapes.

## 2 Materials and methods

### 2.1 Study region

**Figure 1:**
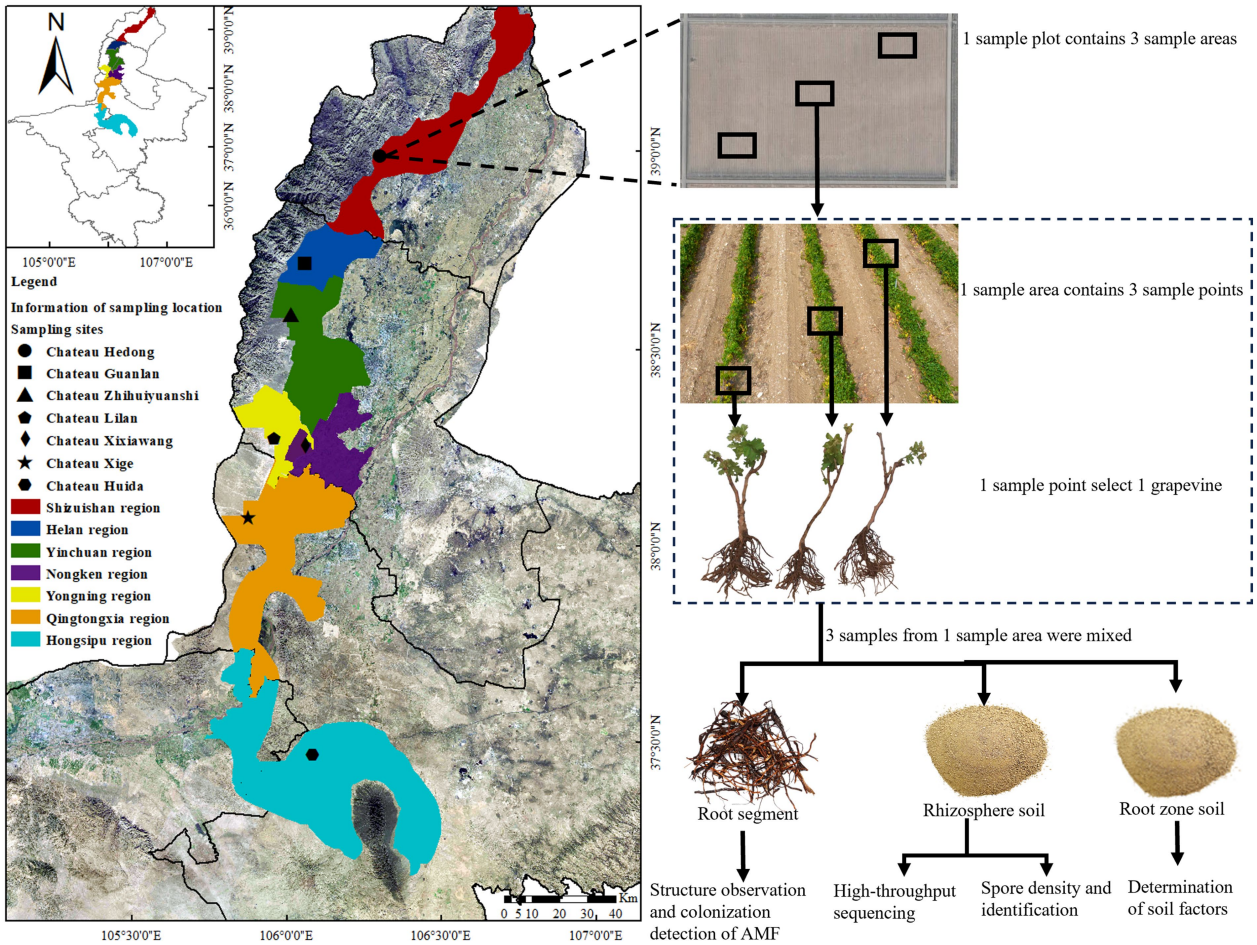
Geographic map of sampling points and sampling scheme.

The study was conducted in a grape-producing area in the EFHM in the Ningxia Hui Autonomous Region of China. This region, located at 37°43′–39°23′N and 105°45′–106°47′E on the western edge of the Yinchuan Plain and with an average altitude of 1,118–1,333 m, is one of China’s most important grape-producing areas (Figure 1). The area experiences a temperate continental arid and semi-arid climate, with daily temperature variations of 12 to 15 °C and annual precipitation of 150–240 mm. The soil in this region is primarily composed of sand, silt, and gravel, with low organic matter content and good aeration and permeability. These unique geographical and environmental conditions are conducive to the growth of wine grapes.

Unlike most wine-producing regions globally, in the western part of China, including the EFHM, the temperature in winter reaches as low as −20 °C. Therefore, to mitigate the risk of grapevine damage due to freezing, it is necessary to prune vines and bury them in the soil in November. The grapevines in this region predominantly consist of self-rooted seedlings, as opposed to grafted rootstocks, which is a key factor in the successful implementation of this burying method. These regions of western China are designated as “buried cold regions”. Based on different factors such as climate, soil type, and altitude, the grape-producing area was divided into seven ecological regions, predominantly cultivating *V. vinifera* cv. Cabernet Sauvignon, Merlot, Syrah, Chardonnay, and Riesling varieties. In this study, representative winery vineyard plots were selected as sample collection points for seven ecological regions. To reduce experimental error, seven Cabernet Sauvignon vineyards with vines aged >8 years were selected. The vineyard locations were Chateau Hedong (HD) in the Shizuishan production area, Chateau Guanlan (GL) in the Helan production area, Chateau Zhihuiyuanshi (ZH) in the Yinchuan production area, Chateau Lilan (LL) in the Yongning production area, Chateau Xixiawang (XXW) in the Nongken production area, Chateau Xige (XG) in the Qingtongxia production area, and Chateau Huida (FHD) in the Hongsipu-production area. The general characteristics of the seven winery plots are shown in Figure 1 and Table 1.

**Table 1:** Soil factors and geographical overview of the study region.

Item	Chateau Hedong (HD)	Chateau Guanlan (GL)	Chateau Zhihuiyuanshi (ZH)	Chateau Lilan (LL)	Chateau Xixiawang (XXW)	Chateau Xige (XG)	Chateau Huida (FHD)
Production areas	Shizuishan region	Helan region	Yinchuan region	Yongning region	Nongken region	Qingtongxia region	Hongsibu region
Management mode	Clean cultivation	Inter-row (inner) grass	Inter-row (inner) grass	Clean cultivation	Clean cultivation	Clean cultivation	Clean cultivation
Latitude and longitude	38°58′53″N,106°18′34″E	38°42′58″N,106°03′48″E	38°34′18″N,106°01′43″E	38°16′41″N,105°58′18″E	38°15′03″N,106°01′47″E	38°04′13″N,105°52′30″E	37°27′57″N,106°05′02″E
Elevation/m	1,118	1,181	1,164	1,153	1,143	1,225	1,333
Soil types	Gravel calcareous chernozem	Gravel calcareous chernozem	Shallow calcareous soil, gravelly sandy loam	40% gravel and 60% clay and calcareous soil	gray desert soil	Light gray calcic soil	Light gray calcic soil
Organic matter (g/kg)	14.94 ± 0.14a	8.13 ± 0.25b	8.27 ± 0.18b	4.28 ± 0.06c	4.38 ± 0.06c	3.01 ± 0.20d	0.84 ± 0.02e
Total nitrogen (g/kg)	0.34 ± 0.00d	0.60 ± 0.00b	0.40 ± 0.00c	0.35 ± 0.00d	0.81 ± 0.02a	0.79 ± 0.03a	0.17 ± 0.01e
Alkaline hydrolysis nitrogen (mg/kg)	72.10 ± 0.23c	86.45 ± 3.85a	81.55 ± 2.68b	46.20 ± 2.80e	54.13 ± 0.93d	33.83 ± 1.67f	46.78 ± 1.28e
Available phosphorus (mg/kg)	16.82 ± 0.32e	22.47 ± 1.82c	46.09 ± 0.22a	19.42 ± 0.65d	45.72 ± 1.53a	34.39 ± 1.07b	21.27 ± 1.14 cd
Available potassium (mg/kg)	88.55 ± 0.34b	73.92 ± 0.00d	81.29 ± 0.79c	89.22 ± 0.34b	90.14 ± 1.70b	67.79 ± 0.68e	113.35 ± 2.94a
pH	8.13 ± 0.01f	8.16 ± 0.00e	8.20 ± 0.01d	8.44 ± 0.01a	8.25 ± 0.00b	8.43 ± 0.01a	8.23 ± 0.00c
Catalase (mg/g)	0.58 ± 0.06d	1.08 ± 0.02a	0.92 ± 0.05b	0.72 ± 0.00c	0.77 ± 0.05c	0.93 ± 0.03b	0.37 ± 0.01e
Alkaline phosphatase (mg/g)	1.34 ± 0.03c	2.57 ± 0.03b	3.09 ± 0.01a	1.33 ± 0.03c	0.62 ± 0.02e	0.30 ± 0.04f	1.14 ± 0.01d
Urease (mg/g)	3.14 ± 0.02e	6.55 ± 0.08a	6.29 ± 0.04b	4.61 ± 0.05c	3.70 ± 0.04d	1.73 ± 0.10f	4.60 ± 0.05c
Invertase (mg/g)	2.13 ± 0.02b	3.33 ± 0.06a	3.23 ± 0.09a	0.77 ± 0.09e	1.48 ± 0.04c	0.59 ± 0.02f	1.25 ± 0.03d

The different letters (a - f) indicate significance at *p* = 0.05 level.

### 2.2 Experimental design and sampling strategy

In August 2022, we collected root segments and rhizosphere soil samples from *V. vinifera* cv. Cabernet Sauvignon wine grapes from seven vineyard plots (HD, GL, ZH, LL, XXW, XG, and FHD). Each plot covered approximately 6.67–8 hm^2^ and the same cultivation and management practices were followed. Before sampling, we conducted preliminary observations by excavating the roots of grape. We found that the roots were primarily distributed within 0–60 cm of the soil surface. Consequently, the sampling range was set at this depth.

At each plot, we established three sampling points (20 × 20 m) using the diagonal sampling method, ensuring each point was more than 100 m apart. After removing the surface gravel and dead weeds, we randomly selected three healthy grapevines at each sampling point. Lateral root segments (diameter: 0.1–0.5 mm and length: 10–100 mm) and rhizosphere soil samples were collected from the 0–60 cm soil layer, within a 0–30 cm radius around the main stem of the grapevines. Nine grape trees were collected from each plot. The soil adhering to the roots was defined as the rhizosphere soil (Zhou et al., 2019). The sampling method was as described by Liu et al. (2022). We used a sterile brush to brush the soil tightly attached to the root surface and then passed it through a 1 mm soil sieve to prevent excess dead root bark litter from entering the sample. After the rhizosphere soil of three vines in each sample point was fully mixed, we retained 5 g soil samples using the quartering method, placed them into 2 mL sterile centrifuge tubes, transported them to the laboratory in a cooling box, and froze them at −80 °C for high-throughput sequencing. The remaining rhizosphere soil was stored at 4 °C for AMF spore identification and spore density detection. Grape root segments were cut using sterile scissors, washed with sterile water, and stored in formalin-aceto-alcohol (FAA) fixative solution (*V* (38% formaldehyde): *V* (glacial acetic acid): *V* (75% alcohol) = 1:1:18) for morphological structure observation and AMF colonization detection. The soil in the plant growth area was defined as the root zone soil (Shi et al., 2019), and 500 g of root zone soil was collected at each sample point for the determination of soil physical and chemical factors. A total of 21 root segment samples, 21 rhizosphere soil samples (HTS), 21 rhizosphere soil samples (spore identification and spore density detection of AMF) and 21 root zone soil samples were collected from 63 grape trees in seven plots, with three replicates for each treatment (Figure 1).

### 2.3 Observation of AMF structure and infection in wine grape roots

Wine grape root AMF colonization was stained according to the methods described previously by Phillips and Hayman (1970). The root segments were taken out from the FAA and cut into 1 cm. For each sample, 100 roots were selected, transferred into 10% KOH at 90 °C for 1 h, acidified with lactic acid for 5 to 10 min, dyed with 0.05% (v/v) acetic acid-ink staining solution (95 mL of white vinegar, 5 mL of Quink ink) for 5 min, and decolored with lactic acid glycerol (the volume ratio of lactic acid and glycerol was 1:1) for 12 h. After decolorization, the samples were observed under an Olympus BX53 microscope (Olympus, Japan) at 200 × magnification, and the root AMF colonization status was assessed using the intersection method (McGonigle et al., 1990)—30 visual fields were examined for each root segment to ensure data representativeness. The root AMF colonization rate and colonization intensity were calculated as follows (Equations 1, 2) (Li et al., 2011; Yan et al., 2014):


(1) $$\begin{array}{l} \text{Colonization rate}\;(\%)=\\ {}[\frac{\begin{array}{l} \text{number of arbuscular fields}+\\ \text{number of vesicular fields}+\\ \text{number of hyphal fields} \end{array}}{\text{total fields}}]\times 100\% \end{array}$$

(2) $$\begin{array}{l} \text{Colonization intensity}(\%)=\\ \frac{\sum (\frac{\text{infected length of each root segment}}{\text{total length of each root segment}})}{\text{total number of infected root segments}}\times 100\% \end{array}$$

### 2.4 Isolation and identification of AMF in wine grape rhizosphere soil

After the rhizosphere soil samples were dried in the shade, AMF spores were isolated through wet sieve precipitation-sucrose centrifugation (Song et al., 2019). The number of spores was observed and recorded under an Olympus BX53 microscope (Olympus, Japan) at 200 × magnification, and the number of AMF spores per 50 g of air-dried soil was counted as the spore density. Morphological features of spores and the phenotypic and histochemical characters of spore wall layers were determined as described by Omar et al. (1978), using a mixture of polyvinyl-lactic acid-glycerin (PVLG) and Melzer’s reagent (1:1, v/v). The preparation of spores for study, determination of color, and photography of spores and mycorrhizal structures were performed as previously described (Blaszkowski et al., 2012). Species identification was performed according to “Manual for the Identification of Va Mycorrhizal Fungi” (Schenck and Perez, 1990) and the latest taxonomic description and pictures provided by the International Culture Collection of (vesicular) Arbuscular Mycorrhizal Fungi (INVAM) on https://invam.ku.edu/ (Sturmer et al., 2021).

### 2.5 Determination of soil factors

Root zone soil samples were sieved through a 1 mm mesh for the determination of soil physicochemical properties, following the method described by Bao (2000): soil organic matter (SOM) was determined via potassium dichromate-ferrous sulfate titration; total nitrogen (TN) via Kjeldahl digestion; alkali-hydrolyzable nitrogen (AN) via alkali diffusion; pH via a PHS-25 (Shanghai Leici, China) precision pH meter; available phosphorus (AP) via molybdenum-antimony anti-colorimetry; and available potassium (AK) via flame photometry.

Reserved root zone soil samples were sieved through a 0.25 mm mesh for soil enzyme activity analysis, following the method described by Guan (1986). Sucrase activity was determined using 3,5-dinitrosalicylic acid colorimetry, expressed as mg glucose per gram of soil after 24 h; urease activity via indophenol blue colorimetry, expressed as mg NH₃-N per 5 grams of soil after 24 h; catalase activity via potassium permanganate titration, expressed as mL of 0.1 N potassium permanganate per gram of soil after 20 min; and alkaline phosphatase activity via sodium phenyl phosphate colorimetry, expressed as mg phenol released per gram of soil after 24 h.

### 2.6 Fungal community identification with Illumina MiSeq sequencing

#### 2.6.1 DNA extraction and PCR amplification

For DNA extraction, microbial DNA was isolated from the prepared soil samples (0.25 g soil per sample) using the Fast DNA^®^ SPIN Kit for Soil (QBIOgene Inc., Carlsbad, CA, United States), following the manufacturer’s protocols with strict adherence to ensure high-quality DNA recovery. For the PCR amplification, nested PCR was conducted to amplify the AMF 18S rRNA. The primer pairs AML1 (5′-ATCAACTTTCGATGGTAGGATAGA-3′) and AML2 (5’-GAACCCAAACACTTTGGTTTCC-3′) (Lee et al., 2008) were used in the first run. The primer pairs AMV4.5NF (5’-AAGCTCGTAGTTGAATTTCG-3′) and AMDGR (5’-CCCAACTATCCCTATTAATCAT-3′) were used in the second run (Van Geel et al., 2014). The reaction volume was 20 μL, consisting of 4 μL 5 × TransStart FastPfu Buffer, 2 μL of dNTPs (2.5 mmol/L), 0.8 μL each forward and reverse primers (5 μmol/L), 0.4 μL TransStart FastPfu Polymerase (2.5 U/μL), 10 ng/L DNA template, and ddH2O to 20 μL. The PCR procedure was as follows: 95 °C for 3 min; 95 °C for 30 s, 55 °C for 30 s, 72 °C for 45 s; and 72 °C for 10 min and 10 °C for holding. The annealing temperature was 55 °C in the first run for 32 cycles and 55 °C in the first run for 20 cycles. DNA quality was checked using 2% agarose gel electrophoresis.

#### 2.6.2 Illumina sequencing

Purified amplicons were pooled in equimolar amounts, and paired-end sequencing (2 × 300 bp) was conducted using an Illumina Nextseq2000 platform (Illumina, San Diego, CA, United States) according to the standard protocols of Majorbio Bio-Pharm Technology Co., Ltd. (Shanghai, China). Sequence read processing was performed using QIIME v.1.9.1 (Wassermann et al., 2019). The raw reads were stored in the NCBI Sequence Read Archive (SRA) database (Accession Number: PRJNA966782) https://www.ncbi.nlm.nih.gov/sra/PRJNA966782.

#### 2.6.3 Bioinformatics analysis

Raw FASTQ files were demultiplexed, quality-filtered using Trimmomatic (Bolger et al., 2014), and merged using FLASH with the following criteria: reads were subjected to truncation at any site that received an average quality score of less than 20 over a 50-base pair sliding window. Primers were matched exactly, with a maximum of two nucleotide mismatches permitted, and reads containing ambiguous bases were removed. Sequences exhibiting a base-pair overlap greater than 10 were amalgamated based on the overlapping sequences. Operational taxonomic units (OTUs) were clustered with a 97% similarity cutoff using UPARSE (v.7.1; http://drive5.com/uparse/) (Edgar, 2013), and chimeric sequences were identified and removed using UCHIME (Edgar et al., 2011). The taxonomy of each 18S gene sequence was analyzed using RDP Classifier (v.2.2, http://sourceforge.net/projects/rdp-classifier/) and the maarjam20220506/AM^1^ 18S database, with a confidence threshold of 95% (Luo et al., 2020).

### 2.7 Data analysis

The alpha diversity indices of the samples, including Simpson even (reflecting community uniformity), Shannon (reflecting community diversity), Chao (reflecting community richness), and PD (reflecting the pedigree diversity of species), were calculated using Mothur software (version v.1.30.2) (Schloss et al., 2009). SPSS 23.0 Duncan’s multiple range test was used for the inter-group test of AMF total colonization rate, colonization intensity, and alpha diversity indexes (Ju et al., 2022). Based on the Qiime software (version 1.9.1) (Bolyen et al., 2019), the beta diversity distance matrix was calculated and the Adonis inter-group difference test results were integrated to construct the principal coordinate analysis (PCoA) diagram (Yan et al., 2022) to determine the differences in AMF among samples. Permutational analysis of variance (PERMANOVA) (Ziegler et al., 2019) was used to decompose the total variance using a semi-metric (Bray-Curtis), analyze the explanatory power of different grouping factors on sample differences, and use a substitution test to analyze the statistical significance of the division. The species composition and relative abundance of each species in each sample were determined at different taxonomic levels and the composition of the dominant species in the different samples was visualized using Pie and Bar diagrams. A Venn diagram (Yan et al., 2022), was used to show the number of shared and unique OTUs of AMF in different groups. LEfSe multi-level species difference discriminant analysis (Segata et al., 2011) was used to evaluate the flora that had a significant impact on sample differences. The free online platform Majorbio I-Sanger Cloud Platform was used to analyze microbiological data.^2^ Soil factors were screened using Variance Inflation Factor (VIF) analysis in the R software vegan package (vsesion2.4.3). VIF analysis was performed on soil factors across different sampling sites, filtering out those with VIF > 10. This screening process was repeated until all selected environmental factors had a VIF < 10. The correlations between soil factors and AMF colonization were analyzed using origin2022, while correlation analysis and mapping between soil factors and AMF communities were performed using RDA in the R software vegan package (version 2.4.3).

## 3 Results

### 3.1 AMF colonization and identification of soil spores of wine grape roots in the EFHM

**Figure 2:**
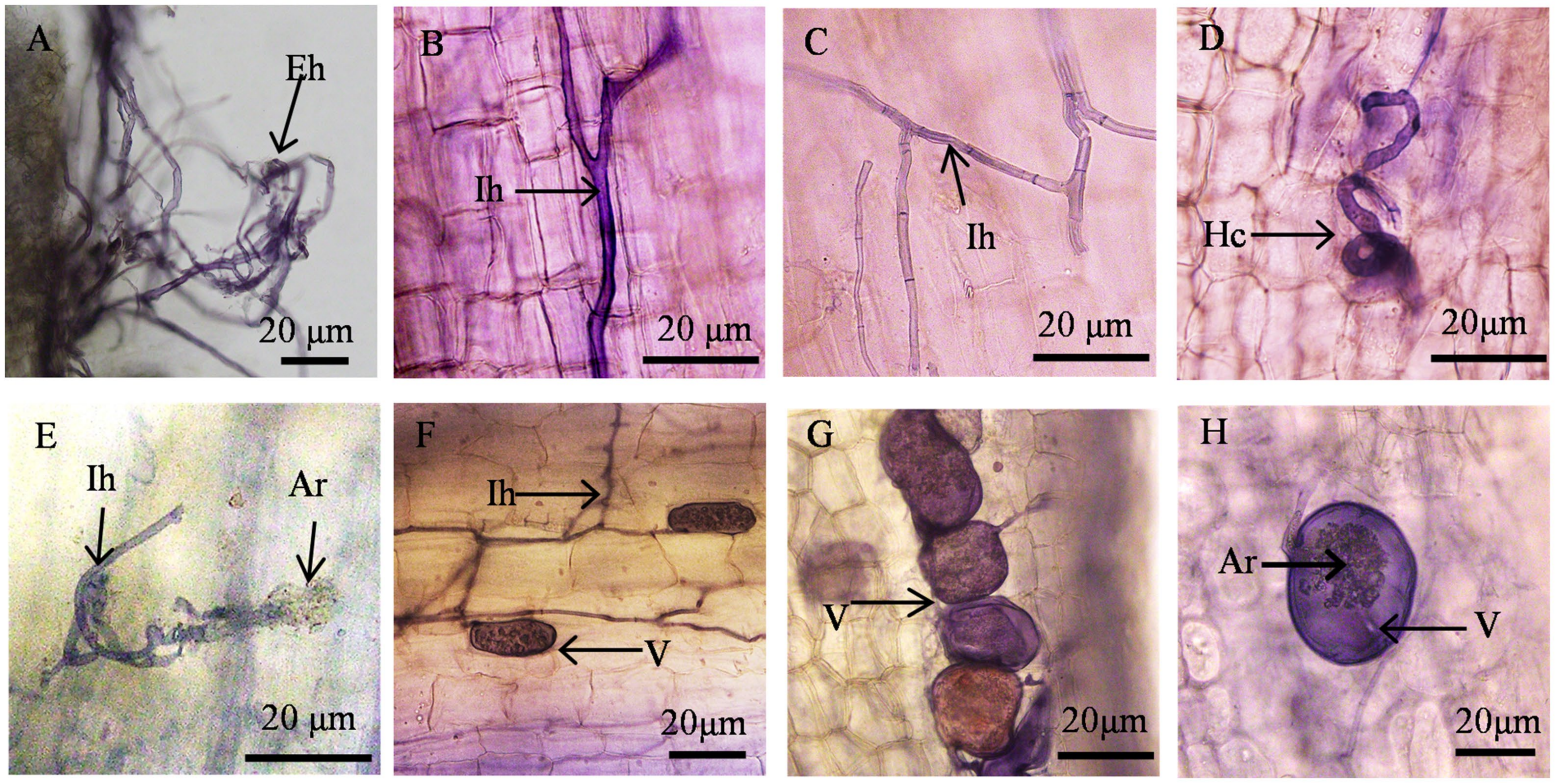
Structure of AMF Colonization in the Root Systems of Wine Grapes. **(A)** Hyphae (Eh); **(B, C)** Internal hyphae (Ih); **(D)** Circular hyphae (Hc); **(E)** Internal hyphae (Ih) and arbuscule (Ar); **(F)** Vesicle (V) and internal hyphae (Ih); **(G, H)** Vesicle (V) and arbuscule (Ar).

AMF invaded the roots of wine grapes, formed a typical AMF structure (arum-type structure), and established a good plant symbiotic relationship (Figure 2). AMF hyphae spread and grew outside the root system of host wine grapes to form extra root hyphae (Figure 2A). It also infected the root system of wine grapes and formed intercellular hyphae in the intercellular spaces of cortical cells. Some intercellular hyphae were straight (Figure 2B), some formed binary branches (Figure 2C), and some were entangled to form mycelial circles (Figure 2D) and intracellular arbuscular structures (Figures 2B–F). Most of the hyphae were non-septate hyphae, although and septate hyphae were formed (Figure 2C). The tops of the hyphae expanded to form vesicles of varying sizes and shapes (Figures 2F–H). The vesicles were mostly round, oval, or irregular in shape and some had inclusions (Figures 2G,H).

**Figure 3:**
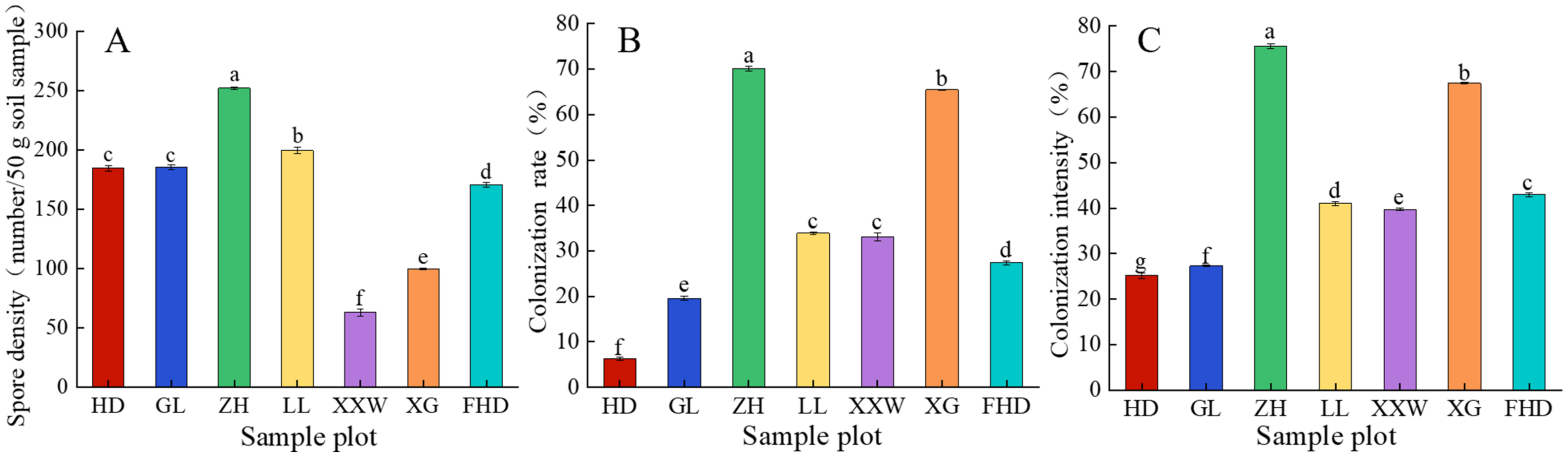
AMF spore density **(A)**, colonization rate **(B)** and colonization intensity **(C)** of grapevines at different sample plots.

As shown in Figure 3A and Supplementary Table S1, the spore density of AMF in the rhizosphere differed by sample plot. The average spore density of AMF was the highest at ZH, at 252 per 50 g, followed by LL, at 199.63 per 50 g. XXW had the lowest, at 63 per 50 g. The root samples of wine grapes in each sample plot were infected with AMF, with colonization rates ranging from 6.28 to 70.04%. Among them, the AMF colonization rate of ZH was the highest (70.04%) and that of HD was the lowest (6.28%; Figure 3B). The AMF colonization intensity in ZH was significantly higher than that observed in the other plots (*p <* 0.05), followed by XG and HD, which were 75.52, 67.41, and 25.14%, respectively (Figure 3C). These data indicate that wine grapes are susceptible to AMF infection.

**Figure 4:**
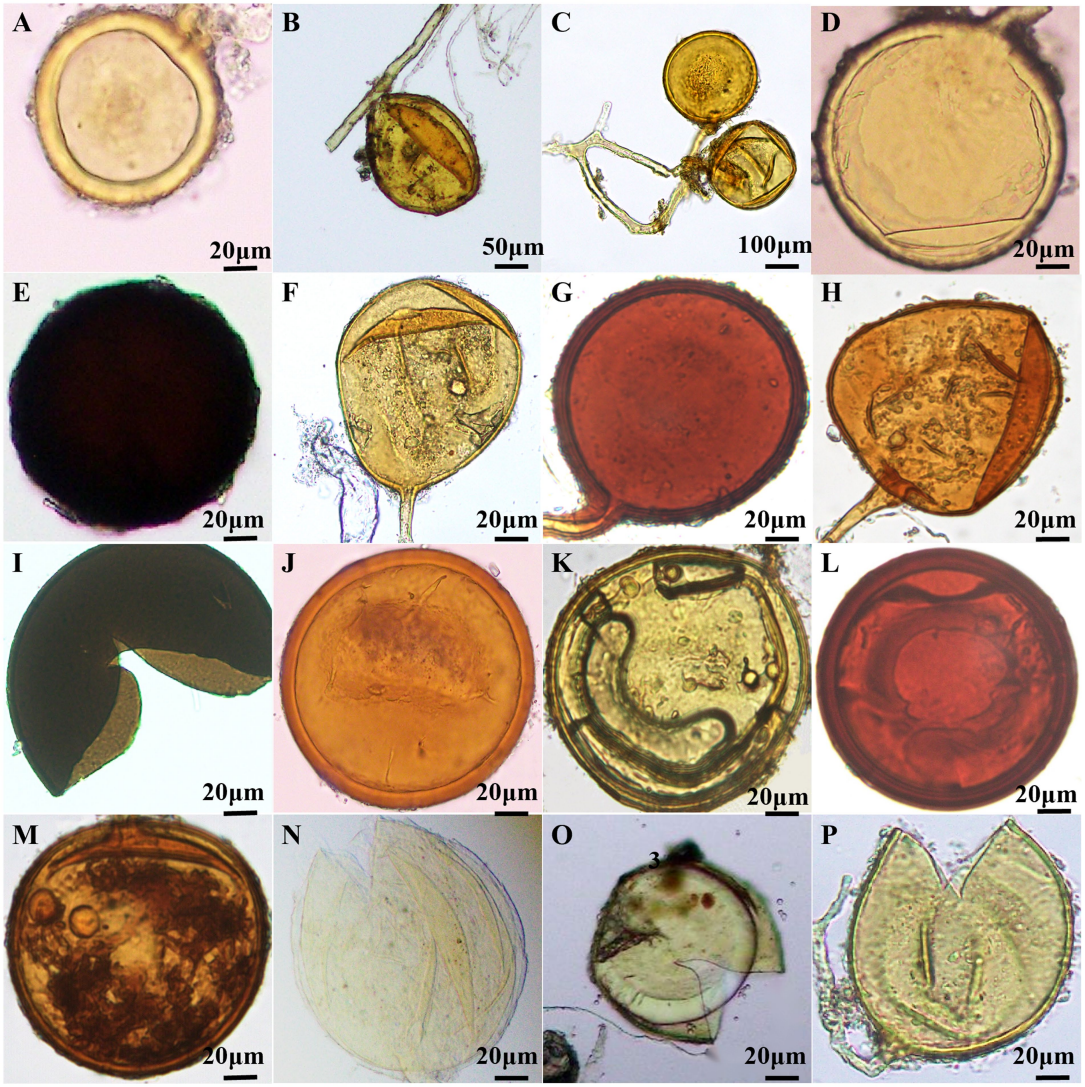
Morphology of AMF spores in wine grape roots. **(A)** *C. claroideum*; **(B)** *C. etunicatum*; **(C)** *Glomus aggregatumv*; **(D)** *G. glomerulatum*; **(E)** *G. melanosporum*; **(F)** *G. pansihalos*; **(G)** *G. reticulatum*; **(H)** *G. Dolichosporm*; **(I)** *G. constrictum*; **(J)** *G. multiforum*; **(K)** *G. clarum*; **(L)** *G. halonatum*; **(M)** *D. etunicatum*; **(N)** *P. brasilianum*; **(O)** *A. dilatata*; **(P)** *S. calospora*.

The morphological characteristics of AMF spores were meticulously documented through microscopic observation, and identified by VA mycorrhizal identification manual. A total of 16 species of AMF belonging to 6 genera were identified (Figure 4; Supplementary Table S2). There were ten species of *Glomus*, two species of *Claroideoglomus*, one species of *Diversispora*, one species of *Paraglomus*, one species of *Acaulospora* and one species of *Scutellospora*. *Glomus* was the dominant genus, and *G. melanosporum* was the dominant species (Supplementary Table S2). *Claroideoglomus, Diversispora* and *Paraglomus* were first discovered in grape plants in Northwest China.

### 3.2 Analysis of AMF community diversity in rhizosphere soil of wine grape in EFHM in Ningxia

#### 3.2.1 AMF *α* diversity in wine grape rhizosphere soil

High-throughput sequencing was performed on the rhizosphere soil from different sample plots and 1,864,896 raw reads were obtained, with a total base number of 559,468,800. After optimizing the data, 1,258,505 valid sequences were obtained, with an average sequence length of 216 bp and base number of 271,975,386 (Supplementary Table S3). Based on a classification level of 97% similarity, a total of 168 OTUs were annotated. The dilution curve (Supplementary Figure S1) showed that, with an increase in the number of sequencing samples, the OTU dilution curve of the rhizosphere soil samples of wine grapes gradually tended to be gentle, indicating that the amount of sequencing data in this experiment was sufficient to fully reflect the AMF species diversity in the rhizosphere soil of different wine grape varieties.

**Figure 5:**
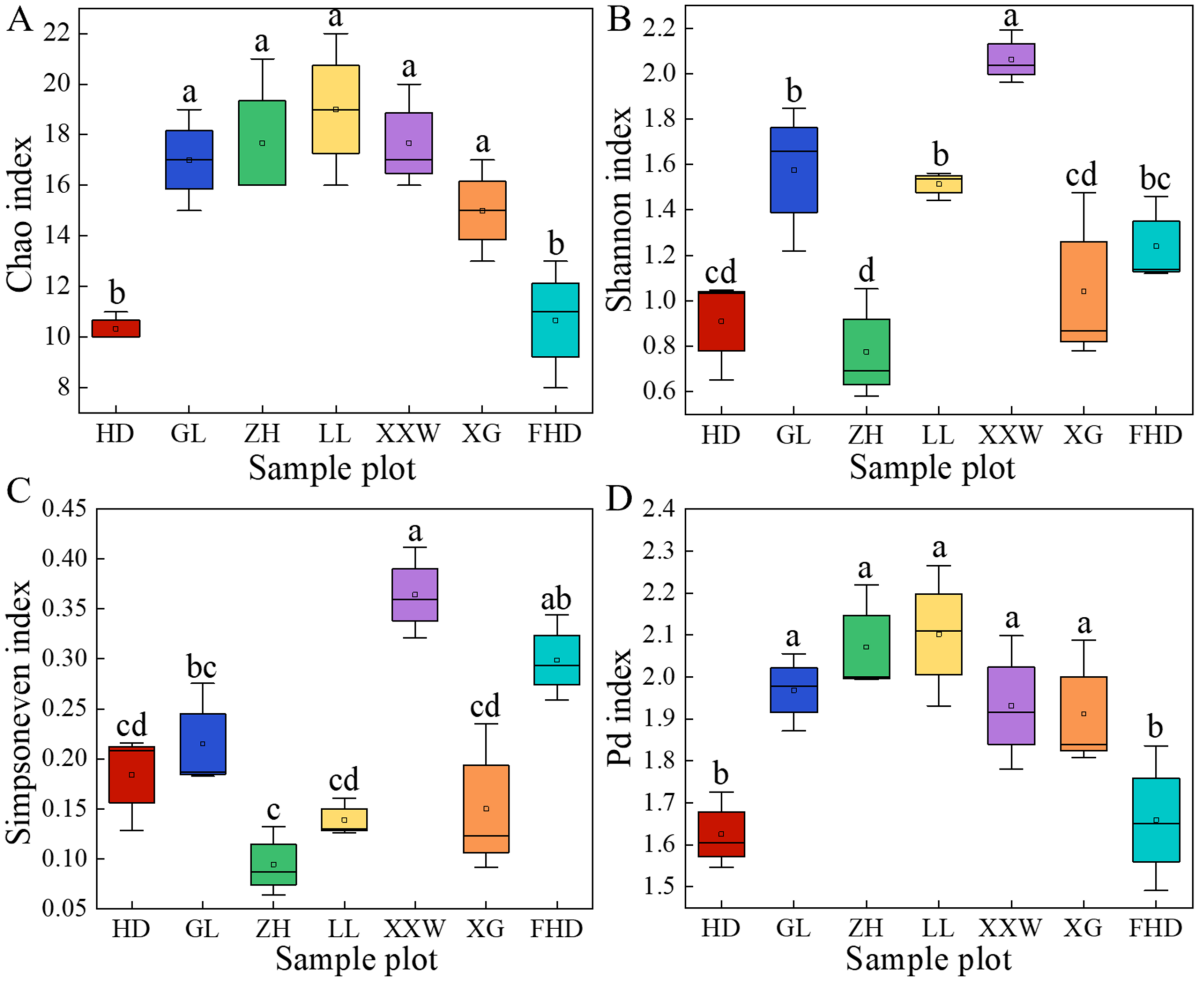
The α diversity index of AMF community in rhizosphere soil of wine grape at species level in different plots. **(A)** The Chao index represents community richness; **(B)** The Shannon index represents community diversity; **(C)** The Simpson evenness index represents community evenness; **(D)** The Pd index (phylogenetic diversity index) represents phylogenetic diversity. The different letters (a - f) indicate significance at *p* = 0.05 level.

As shown in Figure 5, variations in AMF α-diversity were observed across plots. The Chao index of LL was significantly higher than that of the other plots (*p <* 0.05). The ZH plot (17.67) and XXW plot (17.67) ranked second, while the FHD plot had the lowest Chao index (10.33). This indicates that the LL plot had the highest AMF community richness, whereas the FHD plot had the lowest. The Shannon index of the LL plot (2.06) was significantly higher than that of the other plots (*p* < 0.05), with the GL plot (1.58) ranking second and the FHD plot (0.77) having the lowest, indicating that the community diversity of the LL plot was the highest and that of the FHD plot was the lowest. The Simpson-even index (0.36) of the LL plot was the largest, and the Simpson-even index (0.09) of the ZH plot was the smallest, indicating that the uniformity of the LL plot was the highest and that of the ZH plot was the lowest. The highest PD index was observed in the LL plot (2.10), followed by the ZH plot (3.07), whereas the lowest was observed in the HD plot (1.63). The diversity of the AMF lineages in the rhizosphere soil of wine grapes in the seven sample plots was not significantly different (Supplementary Table S4).

#### 3.2.2 Analysis of AMF *β* diversity in wine grape rhizosphere soil

**Figure 6:**
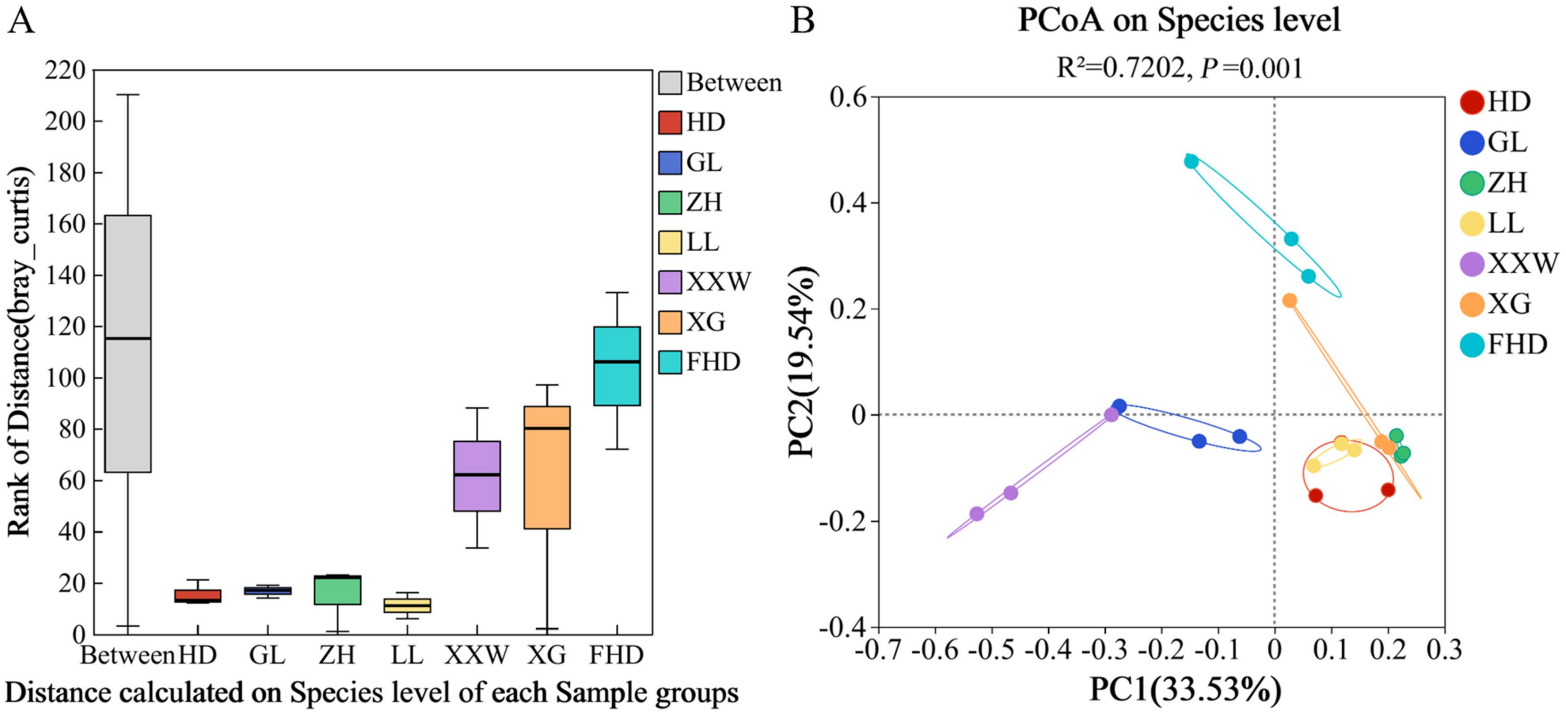
PCoA analysis diagram of soil AMF beta diversity analysis based on the chord algorithm of grapes for different sample plots. **(A)** The X-axis is the distance value within or between groups, the box corresponding to “Between” represents the distance value of the difference between the groups, and the rest of the boxes represent the distance value of the difference within the group; the Y-axis scale represents the size of the distance value. **(B)** The X-axis and Y-axis represent the two selected main coordinate axes, and the percentage represents the interpretation value of the main coordinate axis for the difference in sample composition; The scales of the X-axis and Y-axis are relative distances and have no practical significance; Points with different colors or shapes represent different groups of samples, and the closer the two sample points are, the more similar the species composition of the two samples is.

The β diversity of AMF communities in the rhizosphere soil of wine grapes in different plots was evaluated at the species level, and the analysis results are shown in Figure 6. In the ANOSIM analysis based on the Bray-Curtis distance algorithm (Figure 6A), Clustering was performed on the AMF communities in the rhizosphere soil of wine grapes in the seven sample plots. The variation among samples was significantly higher than that within groups (*p* = 0.001), indicating that the selection of sampling points in this experiment was statistically reliable. Furthermore, PCoA revealed distinct differences in the rhizosphere AMF community of wine grapes from different regions (R = 0.7202, *p* = 0.001; Figure 6B). The PC1 axis explained 33.53% of the data, and the PC2 axis explained 19.54%. The AMF community in the rhizosphere soil of wine grapes in the seven sample plots was divided into three groups. Group I consisted of HD, LL, ZH, and XG. The distribution of samples in these four sample plots was relatively compact, indicating that the similarity of the bacterial community structure was high and the difference in AMF community composition was small. Group II was GL and XXW, and the distance between the two groups was close, indicating that the difference in AMF community structure between the two groups was small. Group III was FHD, which was far from the other six groups, indicating that the similarity of the AMF community structure between FHD and these six groups was low, and the difference was large (Figure 6B).

PERMANOVA analysis (Supplementary Table S5) revealed notably high explanatory power (*R*^2^ = 0.72) and statistical significance (*p* = 0.001) for variations in the microbial community of wine grapes across different sample plots. Furthermore, PERMANOVA on low (HD: 1118 m), medium (XG: 1225 m), and high (FHD: 1333 m) altitudes also demonstrated substantial explanatory power (*R*^2^ = 0.53) and significance (*p* = 0.006) for such variations. Collectively, these results indicate that different sample plots and altitudes are important factors contributing to variations in AMF community within the rhizosphere soil of wine grapes.

### 3.3 AMF community composition of wine grape roots in EFHM in Ningxia

**Figure 7:**
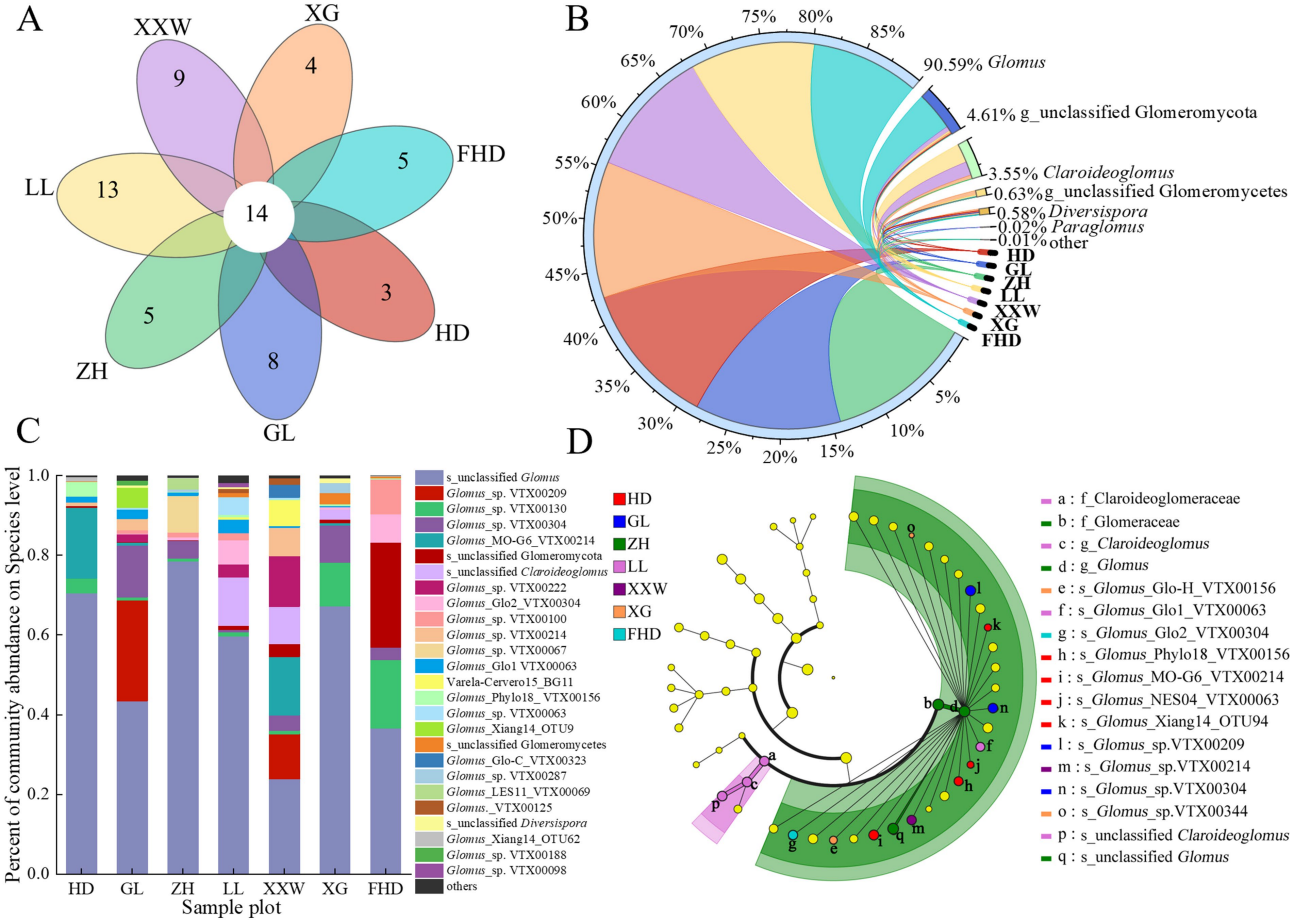
AMF species composition in rhizosphere soil of wine grape in different sample plots. **(A)** Venn diagram of AMF OTU numbers in inter-root soils of “Cabernet Sauvignon” grapes for different sample plots. **(B)** Circos map of horizontal species relationship with AMF genus in the rhizosphere soil of wine grapes (the color of the outer ribbon represents the species, the color of the inner ribbon represents the group from which the sample is distributed, and the length represents the distribution proportion of the sample in a certain species). **(C)** Differences in species composition at the species levels of AMF in inter-root soils of wine grapes at different sample sites (the ordinate is the name of the sample, the abscissa is the proportion of the species in the sample, the columns of different colors represent different species, and the length of the columns represents the proportion of the species). **(D)** LEfSe hierarchical tree analysis of AMF community composition of wine grapes in different plots (the six large circles are phylum, class, order, family and genus from the inside out. Small circle size represents the relative abundance of the species at the taxonomic level. Species with no significant difference are marked in yellow, while species with significant difference are marked in color).

Presently, the membership method is used to define core microbiomes. Membership determines the core microbiome by determining whether there is a common OTU in the host microbiome (Dong et al., 2021). The Venn diagram intuitively reflects the differences in the OTU composition of the AMF community in the rhizosphere soil of different wine grapes and common species. Each sample contained unique OTUs (Figure 7A). The specific OTUs for HD, GL, ZH, LL, XXW, XG, and FHD were 3, 8, 5, 13, 9, 4, and 5, respectively. Fourteen OTUs in the seven plots represented the core microbial group of the soil layer. The shared OTUs were diverse in classification (Supplementary Table S6) but most belonged to *Glomus* and unclassified Glomeromycetes, indicating that the proportion of the same species in the rhizosphere soil samples of wine grapes in the different sample plots was small.

One phylum, three classes, five orders, seven families, seven genera, and 40 species of AMF were identified in the rhizosphere soil of wine grapes from the seven sample plots. At the genus level, the combined proportion outside this genus was relatively low (*p* < 0.01). The five genera with the highest relative abundances were *Glomus* (78.78–99.96%), unclassified Glomeromycota (0.05–26.35%), *Claroideoglomus* (0.20–12.80%), unclassified Glomeromycetes (0.05–2.81%), and *Diversipora* (0.18–1.11%) (Figure 7B). At the species level, unclassified *Glomus* was the dominant species of AMF in the rhizosphere soil of wine grape, with a relative abundance of 23.85–78.55% (Figure 7C). There were substantial disparities in the composition of AMF communities and the prevalence of dominant species in the rhizosphere soil of wine grapes across the diverse sample plots.

LEfSe analysis results indicate that at the species level, the relative abundance of 13 AMFs in the soil of the seven plots showed significant differences (*p <* 0.05) (Figure 7D), mainly belonging to *Claroideoglomus* and *Glomus*. The AMF *Claroideoglomus* was significantly enriched only in LL (*p <* 0.05). The AMF of *Glomus* identified biomarkers in all seven plots, of which HD had the most biomarkers (four biomarkers), followed by XG (two biomarkers) and GL (two biomarkers), while LL, XXW, FHD, and ZH sites each had one *Glomus* AMF identified as a biomarker.

### 3.4 Correlation analysis between soil factors and AMF in different sample plots

All plots had alkaline pH, while other soil nutrients showed some variations (Table 1): HD had the highest organic matter (OM) content (14.94 g/kg, *p* < 0.05). GL exhibited significantly higher contents of alkaline hydrolyzable nitrogen (AN, 86.45 mg/kg), catalase (CAT, 1.08 mg/g), urease (URE, 6.55 mg/g), and sucrase (3.33 mg/g) than other plots (*p* < 0.05). ZH had the significantly highest contents of available phosphorus (AP, 46.09 mg/kg) and alkaline phosphatase (ALP, 3.09 mg/g) (*p* < 0.05). Regarding AN content, XXW ranked the highest (0.81 mg/kg). FHD had the significantly highest available potassium (AK) content (*p* < 0.05). Among these, GL and ZH sites had relatively higher nutrient contents, with more fertile soils. After screening via VIF analysis, the retained soil factors were: soil OM, total nitrogen (TN), AK, AP, pH, and URE—were subjected to correlation analysis (Supplementary Table S7).

**Figure 8:**
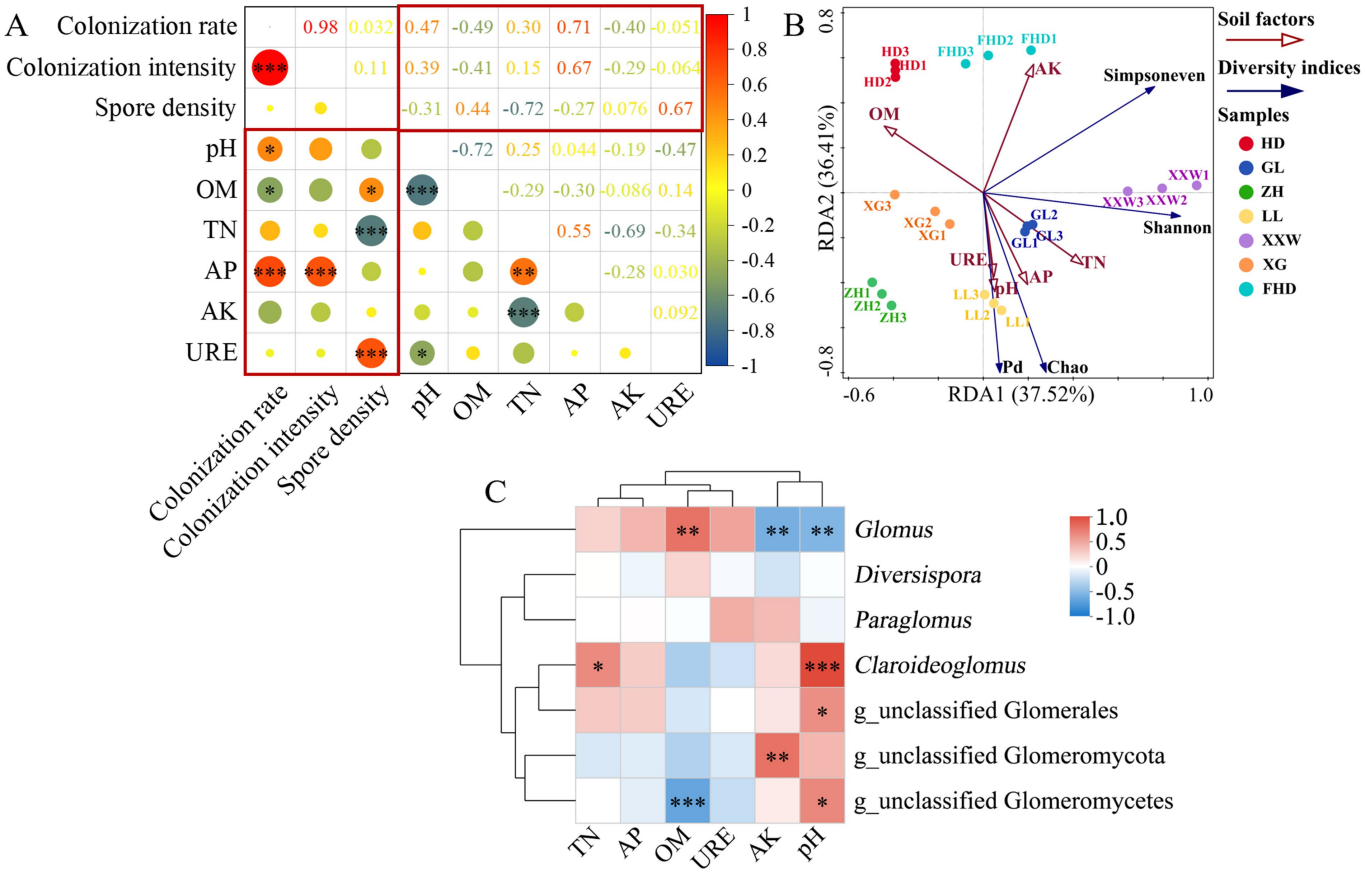
Correlation analysis between environmental factors and AMF. **(A)** Correlation analysis of soil factors and AMF colonization status in wine grapes. (* indicates *p* < 0.05, ** indicates *p* < 0.01, *** indicates *p* < 0.001, OM, organic matter; TN, total nitrogen; AP, available potassium; AK, available phosphorus; URE, urease). **(B)** Analysis of correlation between soil factors and AMF diversity index (species level) in different places (the Angle between the environmental factor arrows represents a positive and negative correlation, acute Angle: positive correlation; Obtuse Angle: negative correlation; Right Angle: no correlation; Projected from the sample point to the arrow of the quantitative environmental factor, the distance from the projection point to the origin represents the relative influence of environmental factors on the distribution of the sample community). **(C)** Spearman’s correlation heat map of AMF community composition (genus level) and soil environmental factors.

Correlation analysis (Figure 8A) revealed the following relationships: AMF colonization rate was significantly positively correlated with soil pH (*p* < 0.05) and highly significantly positively correlated with AP (*p* < 0.001), whereas it was significantly negatively correlated with SOM (*p* < 0.05); colonization intensity showed a highly significant positive correlation with AP (*p* < 0.001); spore density was significantly positively correlated with SOM (*p* < 0.05) and urease (highly significant, *p* < 0.001), but highly significantly negatively correlated with TN (*p* < 0.001).

Redundancy analysis was conducted between the soil factors retained after the VIF analysis and the AMF diversity index (species level) in the wine grape rhizosphere soil. Total nitrogen, available phosphorus, pH, and urease levels positively correlated with AMF diversity (Shannon index), richness (Chao index), and phylogenetic diversity (Pd index). Organic matter was negatively correlated with AMF diversity, richness, evenness (Simpson’s index), and phylogenetic diversity. Available potassium was positively correlated with AMF diversity and evenness and negatively correlated with richness and phylogenetic diversity (Figure 8B). In summary, the principal factors influencing the diversity and abundance of AMF in all environmental variables were soil pH, total nitrogen, available phosphorus, urease, and organic matter.

The perspective of AMF genus *Glomus* was significantly positively correlated with organic matter (*p* < 0.01) and significantly negatively correlated with pH and available potassium (*p* < 0.01). *Claroideoglomus* was significantly positively correlated with pH (*p* < 0.001) and total nitrogen (*p* < 0.05). g_nclassified Glomeromycota was significantly negatively correlated with available potassium (*p* < 0.01). g_unclassified glomerulales was significantly positively correlated with pH (*p* < 0.05). g_nclassified Glomeromycetes was significantly positively correlated with pH (*p* < 0.001) and significantly negatively correlated with organic matter (*p* < 0.05) (Figure 8C).

## 4 Discussion

AMF constitute essential components of the rhizosphere microbial community, forming symbiotic associations with approximately 80% of plants in terrestrial ecosystems (Aseel et al., 2019). In our study, AMF colonized wine grape roots, appearing as clumped mycorrhizae, with total colonization rates ranging from 25.14 to 75.52% and colonization intensities from 6.28 to 70.04%. These findings underscore the ability of AMF to establish symbiotic relationships with wine grape roots. Arbuscular mycorrhiza exhibits major structural classes, including Arum, Paris, and intermediate types (Smith and Smith, 1997). Conversely, A-types are frequently observed in cultivated plants, distinguished by extensive intercellular hyphal growth within the root cortex and the development of terminal arbuscules on intracellular hyphal branches (Smith and Smith, 1997). Our findings indicated that AMF infection in wine grape roots predominantly forms a characteristic arum-type structure, representing a key feature of AMF symbiosis. Given the longstanding cultivation of wine grapes as economically significant fruit trees, this could be one reason for the dominance of this type of infection among AMF structures in wine grape roots.

The methods for identifying AMF include both morphological identification of AMF spores and molecular analysis (Lee et al., 2006; Bidondo et al., 2018; Stevens et al., 2020). Aguilera et al. (2024) identified 15 AMF species from vineyards along a 1,000-km stretch of Chile via spore morphological analysis. They found that the AMF communities in these vineyards were mainly dominated by the genera *Acaulospora, Claroideoglomus, Septoglomus* and *Simiglomus*. Likar et al. (2013) used nested PCR to investigate the colonization and diversity of AMF on grapevine roots in vineyards of karst along the coast of the Adriatic Sea. Their study identified 30 fungal taxa, including the first reported colonization of *G. indicum* in grapevines. In our study, 16 species from 6 AMF genera were identified in 7 plots using the spore morphology identification method. Among these, *Glomus* was the dominant genus, *G. melanosporum* was the dominant species and *Claroideoglomus*, *Diversispora*, and *Paraglomus* were identified for the first time in rhizosphere soils of wine grapes in China. However, spore identification is a time- and energy-intensive process, influenced by regional variations, host plant species, AMF age, and spore morphological variability. Consequently, traditional spore morphological identification poses practical difficulties and fails to truly reflect the natural occurrence of AMF communities (Redecker et al., 2003; Oepik et al., 2009).

High-throughput sequencing provides a more robust and accurate method for studying the community structure and diversity of AMF and can identify AMF in any given root or soil sample without relying on spores (Rodríguez-Echeverría et al., 2017; Rodríguez-Caballero et al., 2018; Faggioli et al., 2019). Moukarzel et al. (2024) used Illumina MiSeq to investigate the composition and abundance of AMF communities in the roots of 12 vineyards in Marburg, New Zealand. The most prevalent AMF genus was *Glomus*, followed by *Entrophospora, Funneliformis, Rhizophagus* and *Diversispora*. In the present study, Illumina MiSeq sequencing identified the fungal communities and divided the AMF OTUs into 1 phylum, 1 class, 5 orders, 7 families, 7 genera, and 40 species. Among them, *Claroideoglomus* and *Glomus* were identified as biomarkers and *Glomus* was the dominant genus in the seven plots, which is consistent with the results of Moukarzel. In addition, *Glomus* is the core genus of AMF in the rhizosphere of wine grapes and plays an active role in plant biological defense. Ye et al. (2022) studied the effects of *G. aggregatum* on the growth, photosynthetic activity, and chlorophyll fluorescence of *Vitis vinifera* L. cv. Ecolly under drought stress, and the results showed that colonization by G. aggregatum increased the dry biomass of shoots and roots, photosynthetic rate, stomatal conductance, and transpiration rate—while decreasing the intercellular CO₂ concentration. Thus, *G. aggregatum* effectively alleviated the negative effects of drought stress on grapevines. Moukarzel et al. (2022) explored the effect of compound microbial agents with *Glomus* on grape black foot disease. The study produced evidence that compound microbial agent with *Glomus* treatments lowered disease incidence at 5 cm and disease severity in vines by 40 to 50% compared to the vines inoculated with the pathogen only. Whether *Glomus* promotes the growth and development of wine grapes in the EFHM requires further investigation.

The diversity and community structure of soil microorganisms are affected by natural factors such as ecological region, climate, soil properties and vegetation. Among them, the ecological region is the main factor affecting soil AMF diversity (Betancur-Agudelo et al., 2021; Rezaee Danesh et al., 2022). Aguilar-Paredes et al. (2024) conducted a study on the diversity of AMF in vineyards across 9 distinct ecological regions within the Mediterranean climate of Chile, and their findings revealed that AMF exhibited a specific geographic distribution, *Acaulospora*, *Scutellospora* sp., *C. etunicatum, Pacispora scintillans* and *Paraglomus* sp. were detected only in the southern valleys, while *Septoglomus* sp. was observed in the Northern and Center Valleys. In the present study, the AMF community structure in the rhizosphere soil of wine grapes from seven ecological regions was divided into three groups. The LEfSe results showed that each ecological region had unique identifying biomarkers. There were some differences in the diversity and abundance of AMF among the different ecological regions. LL and XXW had the highest performance, followed by GL, while FHD had the lowest. We speculate that this may be because LL is located in the Yinchuan Plain, while FHD is located in the Loess Plateau. Changes in topography caused differences in the AMF diversity between the two regions. In addition, the elevation difference of the seven ecological regions in our study exceeded 200 m. PERMANOVA analysis showed that altitude was also a factor that caused AMF diversity and community variation in the rhizosphere soil of wine grapes, which further confirmed that different ecological regions would cause changes in AMF diversity in the ecosystem.

Ecological regions are key macroscale drivers shaping the diversity and community structure of AMF in wine grape rhizospheres. Soil, as the direct habitat for arbuscular mycorrhizal fungi, represents another set of critical regulatory factors. Soil physical and chemical properties and soil enzyme activity, have a direct impact on AMF diversity, community composition, and function (Ren et al., 2021; Song et al., 2021). Correlation analysis showed that soil pH and organic matter were key factors affecting AMF colonization, diversity, and species composition in our study. Soil pH can affect the absorption and utilization of soil elements by plants. An increase in soil pH limits the utilization of soil nutrients and affects the formation and sporulation of AMF (Rillig and Allen, 1999). In our study, the pH values of seven plots ranged from 8.06 to 8.54, with an average of 8.26, indicating that the weakly alkaline soil may be more suitable for the symbiotic relationship between AMF and wine grapes. Correlation analysis showed that pH was significantly positively correlated with the total colonization rate, AMF diversity index and richness index, indicating that the increase of pH was conducive to the formation of AMF, which was consistent with the study of Liu et al. on the correlation between *Hedysarum scoparium* and pH in the northwest desert (Liu et al., 2018). In addition, pH was significantly positively correlated with *Claroideoglomus*, and significantly negatively correlated with *Glomus*, indicating that an alkaline environment was favorable for the growth of *Claroideoglomus*, whereas *Glomus* preferred a neutral and acidic environment. Soil organic matter is a crucial source of nutrients and a significant indicator of soil fertility and quality. Its content directly affects soil fertility (Geng et al., 2023). Correlation analysis showed that organic matter was negatively correlated with total colonization rate and AMF diversity, and positively correlated with spore density and *Glomus*, which was inconsistent with previous studies (Boddington and Dodd, 2000; Wang et al., 2008). A possible reason for this is that AMF spore density and soil organic matter content are only positively correlated within a certain threshold. When the organic matter content exceeded a certain range, this trend stopped or was even reversed, inhibiting the growth of AMF hyphae and vesicle germination.

This study has significant implications for the development and utilization of symbiotic AMF resources in wine grapes in the EFHM of Ningxia, China. Future research could delve deeper into the following areas: identification of beneficial core or key AMF strains to underpin the sustainable cultivation and breeding of grape plants. This entails reducing fertility demands, enhancing nutrient absorption, fortifying resistance to biotic or abiotic stresses, and improving soil structure (Karimi and Noori, 2022). Exploration of how AMF symbiosis influences fruit quality by modulating phenol metabolism, including anthocyanin production, and altering chemical components such as malic acid and tartaric acid. This represents a promising avenue for enhancing grape quality and serves as a potential regulatory resource (Velasquez et al., 2020). Investigation of the impact of AMF on amino acids or volatile compounds in grape plants to enhance the taste, flavor, and aroma of wine. This research can lead to advancements in the wine industry. It is crucial to safeguard the natural mycorrhizal fungal communities in vineyards, emphasizing the importance of preserving and protecting the delicate balance of these beneficial symbionts within the ecosystem.

## 5 Conclusion

High-throughput sequencing technology was used to comprehensively analyze the composition and diversity of AMF communities in the rhizosphere of wine grapes grown in the EFHM. The roots of wine grapes can be colonized by AMF and form a typical structure. The co-evolution of wine grapes and AMF has produced AMF diversity with ecological and environmental specificity. A total of 168 OTUs were detected in the rhizosphere soil of wine grapes in different locations, belonging to five orders, seven families, seven genera, and 40 species of AMF, among which *Glomus* was the dominant genus, indicating abundant AMF resources in the rhizosphere of grapes. Correlation analysis showed that pH and organic matter were key factors affecting AMF richness, diversity, and community composition. This information provides a valuable basis for the protection of AMF communities in the rhizosphere of wine grapes and the commercial cultivation of AMF.

## Data Availability

The datasets presented in this study can be found in online repositories. The names of the repository/repositories and accession number(s) can be found at: https://www.ncbi.nlm.nih.gov/, PRJNA966782.

## Footnotes

^1^ https://www.maarjam.botany.ut.ee/

^2^ http://www.i-sanger.com
