# Supplementary material for: Diversity and drivers of arbuscular mycorrhizal fungi in the rhizosphere soil of wine grape in the eastern foot of Helan Mountain in Ningxia of China
Source: Front Microbiol. 2025 Oct 29;16:1700411. doi: 10.3389/fmicb.2025.1700411 (PMC12605499; doi:10.3389/fmicb.2025.1700411)
Supplement: Supplementary file 1 [file Data_Sheet_1.docx]

**Table S1** AMF infection of wine grapes in different sample plots

| Sampling sites | Colonization rate/% | Colonization intensity/% | Spore density（/50 g soil） |
| --- | --- | --- | --- |
| HD | 6.28±0.29f | 25.14±0.65g | 184.81±2.31c |
| GL | 19.56±0.44e | 27.31±0.23f | 185.56±2.22c |
| ZH | 70.04±0.56a | 75.52±0.55a | 252.22±3.85a |
| LL | 33.94±0.25c | 40.99±0.47d | 199.63±2.57b |
| XXW | 33.17±0.88c | 39.73±0.81e | 62.96±2.80f |
| XG | 65.43±0.85b | 39.73±0.29b | 99.59±0.61e |
| FHD | 27.37±0.43d | 42.91±0.42c | 170.74±2.31d |

**Table S2** Identification results of soil spores of different sample plots at EFHM in Ningxia

| genus | species | Description spore morphological | HD | GL | ZH | LL | XXW | XG | FHD | Note |
| --- | --- | --- | --- | --- | --- | --- | --- | --- | --- | --- |
| *Claroideoglomus* | *C.claroideum* | The spores are mostly round, light yellow to yellow, and the spore wall is two layers, 4.4 ~ 10 μm thick. | **-** | **+** | **+** | **+** | **-** | **+** | **+** | **5** |
|  | *C.etunicatum* | The spores are brownish yellow, subglobose to irregularly shaped, 90-190 μm in diameter, with two layers of spore wall. The conidiophores are 6-10 μm wide, and the conidiophores are blocked by the spore wall. | **+** | **-** | **+** | **+** | **-** | **+** | **+** | **5** |
| *Glomus* | *G.aggregatum* | The spore fruit is light yellow to yellow brown, 250 ~ 500 μm, and the spore wall is one layer, sometimes two layers, 1.5 ~ 4 μm thick. Mycelial wall thickness was 1 ~ 1.5 μm. | **-** | **-** | **+** | **-** | **+** | **-** | **-** | **2** |
|  | *G.glomerulatum* | The spores are spherical to oval, yellow, and the surface of the spores is smooth, 50-150 μm. The conidiospore hyphae are thicker, and the mycelium wall is connected to the inner wall. The mycelium wall is yellow. | **+** | **-** | **-** | **-** | **-** | **-** | **+** | **2** |
|  | *G.melanosporum* | The spores are solitary in the soil. When mature, they are black brown to black, spherical or nearly spherical, 165-250 μm. The spore wall is a single layer, 8-15 μm thick. | **+** | **+** | **+** | **+** | **+** | **+** | **+** | **7** |
|  | *G.pansihalos* | It is solitary in soil, oval or irregular, 110 ~ 200 μm, with yellow to yellowish brown spores, three layers of spore wall, one continuous spore mycelium, mostly upright, light yellow gradually becomes colorless. | **-** | **+** | **-** | **+** | **-** | **+** | **-** | **3** |
|  | *G.reticulatum* | It is solitary in soil, light reddish brown to deep reddish brown, 80 ~ 100 μm in diameter, spherical or subspherical. L1 is 2 μm thick, L2 is 3 μm thick, and L3 is 3 μm thick. | **-** | **+** | **+** | **+** | **+** | **-** | **+** | **5** |
|  | *G.Dolichosporm* | The spores are yellowish brown to reddish brown, obovate or oblong, occasionally irregular, 65 ~ 220 μm. The spore wall is 3 layers. The conidiospore mycelium is yellowish brown, 7 ~ 10 μm wide, with or without septum. The old spores are sometimes blocked by thickening of the wall. | **+** | **+** | **-** | **+** | **-** | **+** | **+** | **5** |
|  | *G.constrictum* | Soil solitary, black, smooth and bright, round or subrounded, 97-220 μm, spore wall 1 layer. | **+** | **+** | **+** | **+** | **-** | **-** | **+** | **5** |
|  | *G.multiforum* | It is solitary in soil, dark yellow to brown, spherical to subglobose, 125-175 μm, spore wall 3 layers, L1 layer transparent, L2 layer close to L1 layer, transparent, disappeared with age, L3 is layered wall, dark yellow to brown. | **+** | **+** | **+** | **-** | **-** | **-** | **+** | **4** |
|  | *G.clarum* | It can be solitary in the soil, spherical to nearly spherical, with a large change in diameter of 60-260 μm. The buns are transparent to light yellow. Most of the spores in the soil have a year-to-year coat, which falls off with age, forming crepes or bulges on the surface of popped purple rice. The spore wall has 2 layers, L1 layer is 5-20 μm, transparent to light yellow, and L2 layer is transparent. | **-** | **-** | **-** | **-** | **-** | **+** | **-** | **1** |
|  | *G.halonatum* | The spores are solitary in soil, yellow to reddish brown, spherical or nearly spherical, 120-210 μm. | **-** | **-** | **+** | **+** | **-** | **-** | **-** | **2** |
| *Diversispora* | *D.etunicatum* | The spores are round or nearly round in the soil, with a diameter of 70-130 μm, yellow or yellowish brown. The surface of the spores is smooth, and the spore wall is two layers. The L1 layer is transparent and perishable, and the L2 layer is yellow to yellowish brown, with a thickness of 3-6.5 μm. Only one layer of laminated wall can be seen on the mature spores, and the spore contents are concentrated and granular. | **+** | **+** | **+** | **+** | **+** | **+** | **-** | **6** |
| *Paraglomus* | *P.brasilianum* | The spores are solitary in the soil, transparent or light gray, spherical to nearly spherical, with a diameter of 70-130 μm. The wall of L1 layer is perishable, the surface of L2 layer has small folds, and the L3 layer is transparent. Conidiospore mycelium : cylindrical or slightly open, mycelium wall transparent to light gray | **-** | **+** | **-** | **-** | **+** | **-** | **+** | **3** |
| *Acaulospora* | *A.dilatata* | The spores are globose or subglobose, with three layers of 100-160 μm spore wall. The outermost layer of L1 is transparent and perishable, with a diameter of 100-140 μm. The L2 and L3 layers are light yellow. | **-** | **-** | **-** | **-** | **+** | **+** | **-** | **2** |
| *Scutellospora* | *S.calospora* | The spores are spherical to oval, 160-280 μm, light yellow, with layered spore wall, 3-5 μm thick, fragile, transparent to light yellow, usually with a slender mycelium. | **-** | **+** | **-** | **+** | **-** | **+** | **+** | **4** |

**Table S3 Statistics of** **Illumina MiSeq high-throughput sequencing output**

| Sample\Info | Seq_num | Base_num | Mean_length | Min_length | Max_length |
| --- | --- | --- | --- | --- | --- |
| FHD1 | 57275 | 12657789 | 221.0002 | 211 | 248 |
| FHD2 | 58053 | 12722347 | 219.1506 | 211 | 281 |
| FHD3 | 47518 | 10246978 | 215.6441 | 212 | 248 |
| GL1 | 67920 | 14618270 | 215.2278 | 211 | 229 |
| GL2 | 64202 | 13811785 | 215.1301 | 211 | 221 |
| GL3 | 62187 | 13375967 | 215.0927 | 210 | 229 |
| HD1 | 63048 | 13589584 | 215.5435 | 209 | 248 |
| HD2 | 62524 | 13469330 | 215.4266 | 211 | 248 |
| HD3 | 61088 | 13152685 | 215.3072 | 211 | 248 |
| LL1 | 109052 | 23592052 | 216.3376 | 211 | 274 |
| LL2 | 54720 | 11851287 | 216.5805 | 212 | 274 |
| LL3 | 52988 | 11436466 | 215.8312 | 212 | 235 |
| XG1 | 50336 | 10871321 | 215.9751 | 211 | 295 |
| XG2 | 51146 | 11024948 | 215.5584 | 212 | 229 |
| XG3 | 51398 | 11075707 | 215.4891 | 212 | 277 |
| XXW1 | 48414 | 10507273 | 217.0296 | 212 | 226 |
| XXW2 | 46674 | 10064687 | 215.638 | 211 | 235 |
| XXW3 | 46436 | 10006782 | 215.4962 | 212 | 234 |
| ZH1 | 70284 | 15176283 | 215.928 | 205 | 227 |
| ZH2 | 68090 | 14680729 | 215.6077 | 209 | 229 |
| ZH3 | 65152 | 14043116 | 215.5439 | 211 | 218 |

**Table S4** Diversity indices of AMF communities in rhizosphere soils of wine grapes at different sample plots

| Sample | chao index | Shannon index | Simpsoneven index | Pd |
| --- | --- | --- | --- | --- |
| HD | 10.33±0.58b | 0.91±0.22cd | 0.18±0.05cd | 1.63±0.09b |
| GL | 17.00±2.00a | 1.58±0.32b | 0.22±0.05bc | 1.97±0.09a |
| ZH | 17.67±2.89a | 0.77±0.25d | 0.09±0.03d | 2.07±0.13a |
| LL | 19.00±3.00a | 1.51±0.06b | 0.14±0.02cd | 2.10±0.17a |
| XXW | 17.67±2.08a | 2.06±0.12a | 0.36±0.05a | 1.93±0.16a |
| XG | 15.00±2.00a | 1.04±0.38cd | 0.15±0.08cd | 1.91±0.15a |
| FHD | 10.67±2.52b | 1.24±0.19bc | 0.30±0.04ab | 1.66±0.17b |

**Table S5** PERMANOVA analysis of AMF microbial community structure in different sites and Altitude of wine grape root soil

| **Characteristics** | **SumsOfSqs** | **MeanSqs** | **F.Model** | ***R^2^*** | ***P*.value** | ***P*.adjust** |
| --- | --- | --- | --- | --- | --- | --- |
| Site | 2.01849 | 0.33641 | 6.0047 | 0.72016 | 0.001 | 0.001 |
| Altitude | 0.51573 | 0.25786 | 3.37098 | 0.52911 | 0.006 | 0.006 |

Note: The *R^2^* value represents the degree of explanation of the grouping factors on the differences of the samples; a larger *R^2^* indicates a higher degree of explanation of the differences by grouping; a p-value less than 0.05 indicates a high confidence level of this test. Significant differences are highlighted in bold (*P*<0.05).

**Table S6** The species composition of common OTUs

| OTU | Order | Family | Genus | Species |
| --- | --- | --- | --- | --- |
| OTU110 | o__Glomerales | f__Glomeraceae | g__*Glomus* | s__unclassified *Glomus* |
| OTU56 | o__Glomerales | f__Glomeraceae | g__*Glomus* | s__unclassified *Glomus* |
| OTU55 | o__Glomerales | f__Glomeraceae | g__*Glomus* | s__unclassified *Glomus* |
| OTU157 | o__Glomerales | f__Glomeraceae | g__*Glomus* | s__unclassified *Glomus* |
| OTU105 | o__Glomerales | f__Glomeraceae | g__*Glomus* | s__unclassified *Glomus* |
| OTU64 | o__unclassified Glomeromycetes | f__unclassified Glomeromycetes | g__unclassified Glomeromycetes | s__unclassified Glomeromycetes |
| OTU18 | o__Glomerales | f__Glomeraceae | g__*Glomus* | *Glomus* sp._VTX00130 |
| OTU2 | o__Glomerales | f__Glomeraceae | g__*Glomus* | s__unclassified *Glomus* |
| OTU93 | o__Glomerales | f__Glomeraceae | g__*Glomus* | s__unclassified *Glomus* |
| OTU11 | o__Glomerales | f__Glomeraceae | g__*Glomus* | s__unclassified *Glomus* |
| OTU83 | o__Glomerales | f__Glomeraceae | g__*Glomus* | *Glomus* Phylo18_VTX00156 |
| OTU28 | o__Glomerales | f__Glomeraceae | g__*Glomus* | s__unclassified *Glomus* |
| OTU126 | o__Glomerales | f__Glomeraceae | g__*Glomus* | *Glomus* GloH_VTX00156 |
| OTU129 | o__Glomerales | f__Glomeraceae | g__*Glomus* | s__unclassified *Glomus* |

**Table S7** Soil factor VIF values before and after screening

| Soil nutrients | OM | TN | AN | AK | AP | pH | CAT | ALP | URE | INV |
| --- | --- | --- | --- | --- | --- | --- | --- | --- | --- | --- |
| VIF value before screening | 46.37 | 171.6 | 120.56 | 25.03 | 56.81 | 110.86 | 93.94 | 563.44 | 182.7 | 387.66 |
| VIF value after screening | 6.49 | 5.72 | \ | 4.96 | 1.71 | 6.45 | \ | \ | 2.76 | \ |

Note: OM: Organic matter , TN: Total nitrogen , AN: Alkali-hydrolyzable nitrogen, AK: Available potassium, AP: Available phosphorus, CAT: Catalase, ALP: Alkaline phosphatase, URE: Urease, INV: Invertase


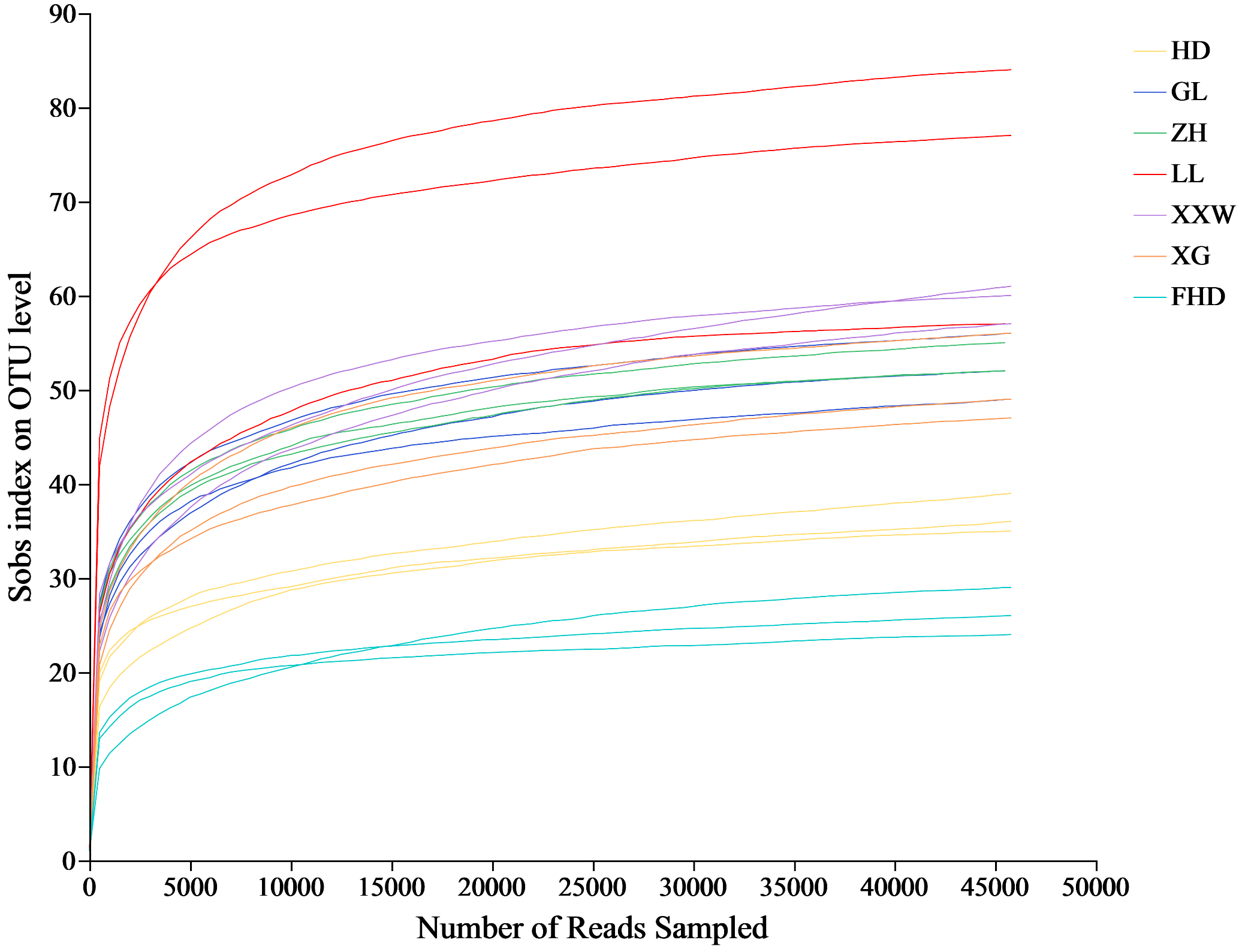


**Figure S1.** Rarefaction curve of AMF in rhizosphere soil of wine grape in different sample plots
